# Avoidable mortality across Canada from 1975 to 1999

**DOI:** 10.1186/1471-2458-6-137

**Published:** 2006-05-23

**Authors:** Paul D James, Doug G Manuel, Yang Mao

**Affiliations:** 1 Institute for Clinical Evaluative Sciences, 2075 Bayview Avenue, Toronto, Canada; 2 The Department of Epidemiology and Community Medicine, University of Ottawa, 451 Smyth Road, Ottawa, Canada; 3 The Department of Public Health Sciences, Faculty of Medicine, University of Toronto, 155 College Street, Toronto, Canada; 4 Surveillance and Risk Assessment Division, Centre for Chronic Disease Prevention and Control, Population and Public Health Branch, Health Canada, Tunney's Pasture, Ottawa, Canada

## Abstract

**Background:**

The concept of 'avoidable' mortality (AM) has been proposed as a performance measure of health care systems. In this study we examined mortality in five geographic regions of Canada from 1975 to 1999 for previously defined avoidable disease groups that are amenable to medical care and public health. These trends were compared to mortality from other causes.

**Methods:**

National and regional age-standardized mortality rates for ages less than 65 years were estimated for avoidable and other causes of death for consecutive periods (1975–1979, 1980–1985, 1985–1989, 1990–1994, and 1995–1999). The proportion of all-cause mortality attributable to avoidable causes was also determined.

**Results:**

From 1975–1979 to 1995–1999, the AM decrease (46.9%) was more pronounced compared to mortality from other causes (24.9%). There were persistent regional AM differences, with consistently lower AM in Ontario and British Columbia compared to the Atlantic, Quebec, and Prairies regions. This trend was not apparent when mortality from other causes was examined. Injuries, ischaemic heart disease, and lung cancer strongly influenced the overall AM trends.

**Conclusion:**

The regional differences in mortality for ages less than 65 years was attributable to causes of death amenable to medical care and public health, especially from causes responsive to public health.

## Background

Rising health care costs and concern regarding the performance of health services has led to the development of various indicators of health care quality. In 1976, the Working Group on Preventable and Manageable Diseases proposed using "unnecessary untimely deaths" as an indicator of medical care quality [1]. The Working Group presented lists of conditions for which deaths may be amenable if appropriate medical care is provided in a timely fashion. The 'avoidable' mortality (AM) concept has since been applied in various investigations examining differences in mortality between regions and over time.

Temporal trend analyses have demonstrated remarkable declines in AM over the past decades that were much more pronounced when compared to mortality from other causes [2-5]. In the Netherlands, Mackenbach et al[6] demonstrated that declines in mortality from avoidable causes of death were preceded by the introduction of successful medical interventions for those causes. These observations have been used to argue that improvements in medical care have contributed to mortality declines. Following this reasoning, regional comparisons of AM have been conducted in order to highlight areas with excess mortality and stimulate further inquiry so that appropriate measures can be taken [7,8]. More recently, the AM concept has been extended to include causes of death amenable to public health [9,10]. AM has been proposed as a population-based performance measure for Canada's health care system[11,12].

The objective of this study was to examine mortality trends from avoidable causes of death in Canada. We examined mortality from 1975–1979 to 1995–1999 in five geographic regions of Canada. These trends were compared to mortality from other causes of death not classified as avoidable. In addition, we investigated the contribution of each avoidable condition to all-cause mortality in Canada.

## Methods

Mortality data were obtained from the Canadian Mortality Database. The selection of avoidable causes of death was based on disease and age groups proposed by the Concerted Action of the European Community on "Avoidable Deaths" with the addition of lung cancer and injuries (see Table 1)[13,11]. The conditions were categorized as causes amenable to medical care or public health. Deaths amenable to medical care are those for which it is reasonable to expect deaths to be averted after the condition has developed. This list includes causes such as appendicitis and asthma, where the medical nature of the intervention is evident. This list also includes causes such as cervical and breast cancer, where deaths are preventable through early detection and effective treatment. Deaths amenable to public health are those for which medical care may be less effective for treating the condition, but where there are interventions that are known to prevent the condition from occurring. This group includes deaths from lung cancer, which are mainly amenable to lifestyle changes (mainly tobacco use), and deaths from injuries, which are influenced by social safety programs and legislative measures. Ischaemic heart disease (IHD) was categorized separately because both medical care and public health can contribute to reducing deaths from IHD [14]. Evidence demonstrating that the causes of death are amenable to health care interventions are referenced. The deaths that were not classified as avoidable were classified as deaths from other causes.

The data analyses were restricted to deaths under 65 years of age, since health care can have the greatest impact on preventing deaths in this group. Deaths from other causes were calculated as the difference between all-cause deaths and avoidable deaths. There are various conditions that are amenable to health care but have not previously been included in avoidable deaths classification lists. Although this is partly due to data availability and/or accuracy issues, this also reflects differences in opinions regarding the preventability of certain causes of death as well as the role of public health and medical care in avoiding deaths[15,16]. For this study, causes of death were selected according to an established and widely disseminated list of avoidable deaths created by the European Community.

The trends in age-standardized mortality from avoidable and other causes of death were estimated for five geographic regions of Canada for consecutive periods (1975–1979, 1980–1985, 1985–1989, 1990–1994, and 1995–1999). The Atlantic region included the provinces Prince Edward Island, Newfoundland, Nova Scotia and New Brunswick. The Prairies region consisted of the provinces Manitoba, Saskatchewan and Alberta. Age-standardized mortality rates (ASMRs) were derived using the direct method with Canada 1991 as the reference population.

Mortality trends are presented with 95% confidence intervals assuming that the number of deaths in each age group follow a Poisson distribution.

## Results

**Figure 1:**
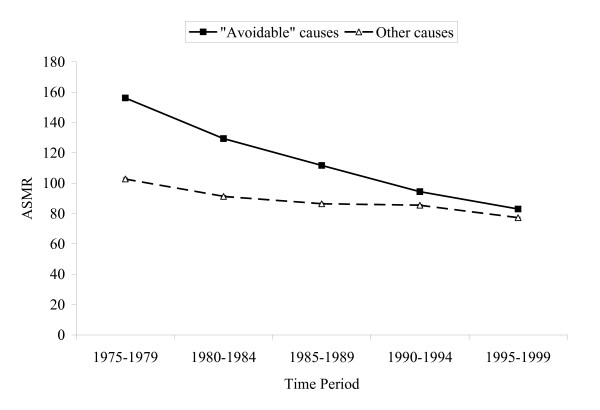
**Mortality from 'avoidable' and other causes in Canada from 1975 to 1999**. Abbreviation: ASMR = age-standardized mortality rate (per 100,000 population).

Figure 1 shows the avoidable and other cause mortality trends in Canada from 1975–1979 to 1995–1999. During this time, mortality from avoidable and other causes decreased. However, the decline in AM (46.9%) was more pronounced from 1975–1979 to 1995–1999 compared to mortality from other causes (24.9%).

**Figure 2:**
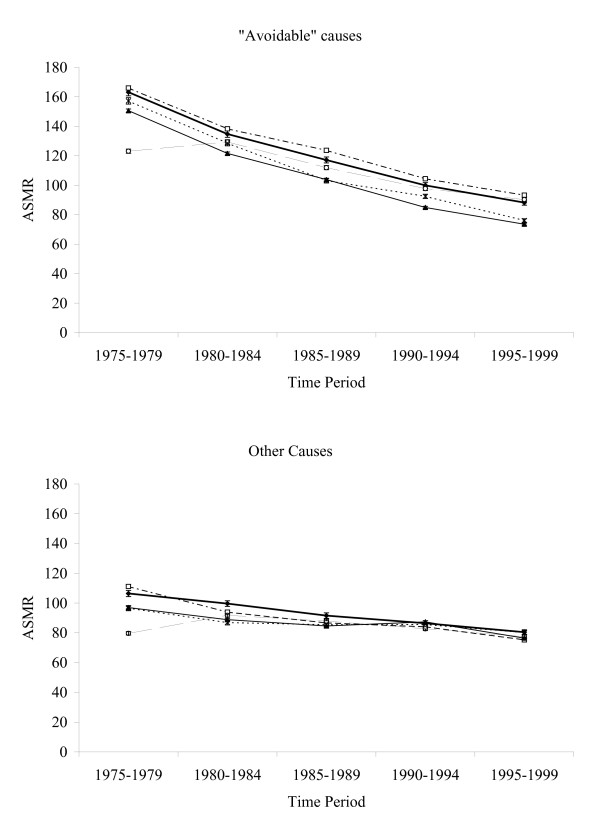
**Time trends for regional mortality from 'avoidable' and other causes from 1975 to 1999**. Abbreviation: ASMR = age-standardized mortality rate (per 100,000 population).

Figure 2 illustrates avoidable and other cause mortality trends in the five regions of Canada. For AM, there was a persistent difference between the highest and lowest Canadian region since 1980–1984. Ontario and British Columbia had consistently lower AM trends than the other regions. For other causes, there was a narrowing of provincial differences over the study period. By 1990–1994 provincial differences for other causes was small.

Table 2 and Table 3 describe the mortality trends from each avoidable cause of death for males and females in Canada and the proportion of all-cause mortality under 65 years of age attributable to each avoidable cause. With the exception of lung cancer, mortality from these causes decreased from 1975–1979 to 1995–1999. The relative decrease in mortality from avoidable causes from 1975–1979 to 1995–1999 was similar between males and females. Lung cancer mortality increased by 79.7% in females but decreased from 1980–1984 onward in males for an overall decrease of 21.5%.

From 1975–1979 to 1995–1999, the proportion of all-cause mortality attributable to avoidable causes of death decreased for males (63.3% to 52.6%) and females (54.4% to 50.2%). During 1995–1999, the avoidable causes that contributed the most to all-cause under 65 mortality were injuries, ischaemic heart disease and lung cancer, together contributing 41.3% to all-cause mortality. For females, breast cancer accounted for 10.6% of under 65 mortality during 1995–1999. Hypertension and cerebrovascular disease and perinatal mortality also contributed considerably to all-cause mortality during 1995–1999 (3.0% and 2.3%, respectively).

**Figure 3:**
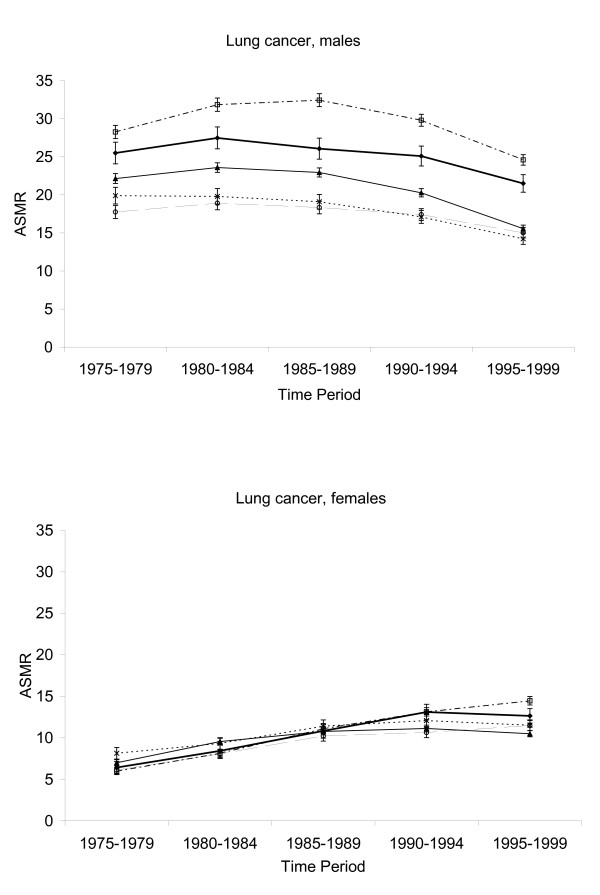
**'Avoidable' mortality from lung cancer in Canada from 1975 to 1999. **The age-groups considered for each cause of death are presented in Table 1. Abbreviation: ASMR = age-standardized mortality rate (per 100,000 population).

**Figure 4:**
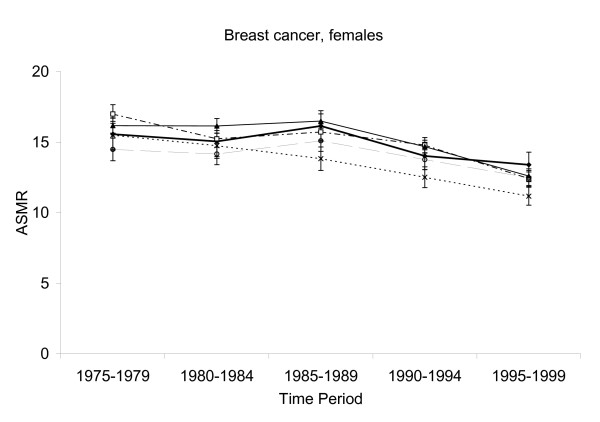
**'Avoidable' mortality from breast cancer in Canada from 1975 to 1999**. The age-groups considered for each cause of death are presented in Table 1. Abbreviation: ASMR = age-standardized mortality rate (per 100,000 population).

**Figure 5:**
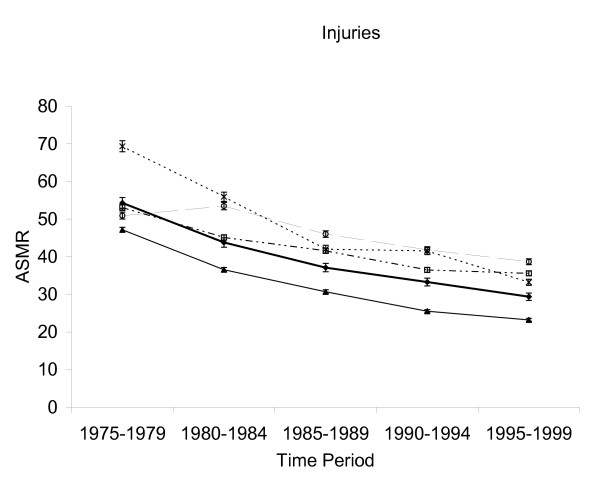
**'Avoidable' mortality from injuries in Canada from 1975 to 1999**. The age-groups considered for each cause of death are presented in Table 1. Abbreviation: ASMR = age-standardized mortality rate (per 100,000 population).

**Figure 6:**
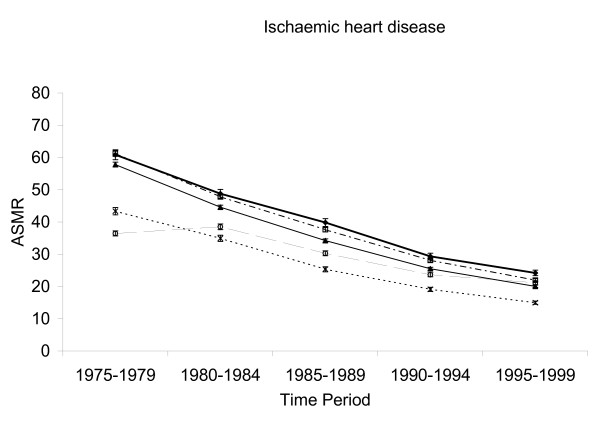
**'Avoidable' mortality from ischaemic heart disease in Canada from 1975 to 1999**. The age-groups considered for each cause of death are presented in Table 1. Abbreviation: ASMR = age-standardized mortality rate (per 100,000 population).

**Figure 7:**
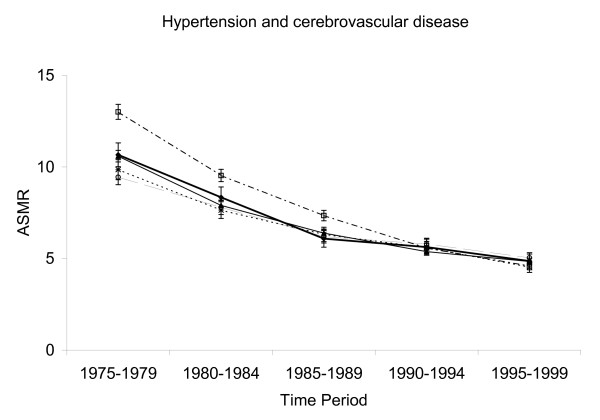
**'Avoidable' mortality from hypertension and cerebrovascular disease in Canada from 1975 to 1999**. The age-groups considered for each cause of death are presented in Table 1. Abbreviation: ASMR = age-standardized mortality rate (per 100,000 population).

**Figure 8:**
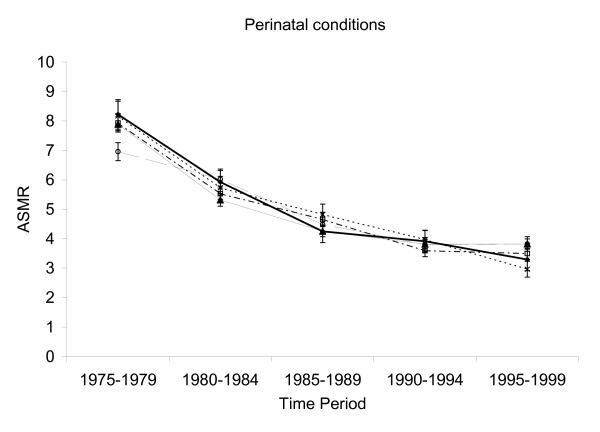
**'Avoidable' mortality from perinatal conditions in Canada from 1975 to 1999**. The age-groups considered for each cause of death are presented in Table 1. Abbreviation: ASMR = age-standardized mortality rate (per 100,000 population).

Figures 3, 4, 5, 6, 7 and 8 present the time trends for mortality from the avoidable causes of death that contributed at least 1% to all-cause mortality under 65 years of age in Canada during 1995–1999. Cause-specific mortality for both sexes was combined, except for deaths from lung cancer (figure 3) and breast cancer (figure 4). The largest regional differences were for injuries (figure 5) and lung cancer (males). Lung cancer for women was the only avoidable cause where mortality differences had increased over time, with increasing mortality in the Quebec and Prairies regions compared to decreasing mortality since 1990–1994 in all other regions. Large differences in regional mortality from ischaemic heart disease (figure 6) continued throughout the study period, although these differences narrowed over time.

## Discussion

This study shows that, while AM has decreased considerably from 1975 to 1999 and regional differences in AM have narrowed, there continues to exist important regional disparities in AM across Canada. Consistently lower AM trends were observed in Ontario and British Columbia – the regions with the highest life expectancy. Ontario experienced the lowest mortality from injuries. British Columbia experienced the lowest mortality from IHD. Both Ontario and British Columbia shared the lowest lung cancer mortality experience with the Prairies. By 1995–1999, these regions showed only small differences in mortality from other causes.

Injuries leading to death include falls, motor vehicle accidents, drowning, burns, bicycle crashes and suicide. Since deaths from these causes can potentially be prevented, various Canadian authors have advocated for the development of co-ordinated injury prevention and safety promotion policies [17,18]. Surveillance and research aimed at determining risk factors associated with injuries in Canada have also been recommended[19]. Bicycle safety legislation represents a good example of a successful intervention for reducing injuries. Using data from the Canadian Institute for Health Information, Macpherson et al [20] demonstrated that bicycle-related head injury rates were found to decrease significantly (45% reduction) in provinces where bicycle helmet legislation had been adopted compared to those provinces and territories where it had not been adopted (27% reduction).

Regional variations in deaths from ischemic heart disease have been shown to be associated with differences in the prevalence of smoking, obesity, hypertension and physical inactivity [21,22]. Thus future efforts aiming to reduce the regional burden of heart disease should include individual and community-based strategies targeting the reduction of these risk factors. Modifying these risk factors also offers the potential for even larger gains in reducing the total burden of disease because heart disease risk factors are also associated with deaths from other chronic conditions, such as diabetes and cancer[23].

Tobacco use accounts for the majority of deaths from lung cancer. Trends in lung cancer mortality reflect the smoking behaviour of the population two decades earlier. Among women, smoking rates peaked in the 1970s and lung cancer deaths continued to increase during the 1990s. Among men, however, smoking rates peaked in the 1960s and lung cancer death rates peaked in the 1980s, falling consistently since then[24].

In addition to traditional risk factors, broader determinants of health, such as income status, employment and education have been shown to be associated with mortality from injuries, IHD and lung cancer[21,22,17,25]. For example, Filate et al[21] have shown that regional death rates from ischaemic heart disease in Canada are significantly associated with regional differences in postsecondary education, unemployment rate and low income rate. Brownell, Frisen and Mayer[17] demonstrated that geographic variations in childhood injury mortality and hospitalizations in Manitoba were strongly influenced by regional differences in neighbourhood income.

A common characteristic of these causes is the potential contribution of public health in their control. Injuries, along with tobacco-related causes of death, may be the most significant deaths that could be prevented through public health initiatives in Canada [11]. Wigle et al [26] estimated that about half of the annual premature deaths in Canada may be avoidable through the control of smoking, diet, elevated serum cholesterol, hypertension, diabetes, and alcohol. The notable regional mortality disparities related to these causes present a warning signal to the Canadian health care system and warrants further investigation.

AM can be considered a useful tool that can provide insights into health care and indicate where further research is warranted [1,27,28]. As others have stated, several characteristics of AM suggest that it is a useful measure of health care performance[29,30]. First, AM one of the few measures that can be measured over time and for different regions both nationally in Canada and internationally. Second, death is an important outcome. This study's findings support the use of AM as a performance measure, particularly when used to compare health regions in Canada. We show that AM in Canada appears to be a surprisingly sensitive measure – one that has considerable variation over time and across regions, and that it likely reflects observed underlying mortality risks that are amenable to public health and health care interventions.

However, AM trends should be interpreted with caution. The definition of avoidable deaths varies between studies and it has recently been shown that observed AM trends are influenced by the list of avoidable deaths applied[31]. In addition, in this study there was a large reduction in mortality not defined avoidable. Many deaths from these conditions were likely amenable to health care interventions. We elected to replicate a previously established list of avoidable deaths to reduce the potential bias of selecting causes of death that would favour one region over another. Future studies should include standard lists of avoidable deaths that are periodically updated as proven health care interventions are effectively introduced.

The AM concept implies that deaths from the selected avoidable causes could have been avoided by optimum health care alone. This is questionable as mortality can be viewed as a long-term outcome that is affected by many factors, including disease incidence, disease severity and risk factors. Additionally, it is difficult to clearly attribute AM trends to specific aspects of health care. It is also important to note that some death that were classified as avoidable may not have been preventable due to factors such as the presence of co-morbidity or treatment complications[13,14]. A meaningful association between AM levels and health care performance and accessibility is lacking[32,30], and thus the validity of AM as an indicator of health care effectiveness is unproven. Furthermore, there are many important functions of the health care services (such as reducing avoidable morbidity and improving quality of life) that cannot be measured using mortality statistics [29,27].

It has been suggested that differences in medically amenable mortality rates may reflect differences in disease incidence. Some authors have attempted to address this potential limitation. For example, Treurniet et al[33] found that regional differences in medically amenable mortality in the Netherlands persisted after adjusting for incidence variations. Some authors have argued that AM trends should not be adjusted for disease incidence or risk factors, since they related to the population's health needs and are within the responsibility of the health system[27].

Future research should seek to understand the relationship between AM levels and health care interventions. Thus far, the association between AM and health care variables have has been characterized as weak and inconsistent[34]. However, the studies conducted in this area used ecological data and considered crude measures of health care. Further, in many countries, mortality trends are taken into account when allocating health care resources. As suggested by Mackenbach et al[34], individual-level data will be necessary in order to determine whether avoidable deaths can be linked to deficiencies in the organization, quality or accessibility of health care services.

Setting age-restrictions for each cause of death is a crude approach to defining avoidable deaths. A useful alternative approach would be to perform sensitivity analyses by comparing trends using alternative age-limits. Furthermore, calculating the proportion of avoidable deaths that are attributable to specific health care interventions may provide more meaningful information for health care decision-makers regarding which interventions would have the greatest impact on improving health and reducing inequalities. For example, in New Zealand, Tobias and Jackson [35] weighted cause-specific mortality trends by the extent to which deaths could be avoided through primary, secondary and tertiary interventions. This expansion of the AM concept can be used to estimate the potential contribution of specific health care interventions to future population health gain.

## Conclusion

Although regional AM differences across Canada have decreased, there continues to exist important regional AM disparities that are mainly attributable to causes of death (injuries, IHD and lung cancer) mainly preventable through public health initiatives.

## Competing interests

The author(s) declare that they have no competing interests.

## Authors' contributions

Paul James had full access to all of the data in the study and takes responsibility for the integrity of the data and the accuracy of the data analysis. Below is a summary of the authors' contributions to this manuscript:

Study concept and design: PJ, DM, YM

Acquisition of data: DM, YM

Analysis and interpretation of data: PJ, DM

Drafting of the Manuscript: PJ, DM, YM

Critical revision of the manuscript for important intellectual content: PJ, DM, YM

Statistical analysis: PJ, DM, YM

Obtained funding: DM, YM

Administrative, technical, or material support: DM

Study supervision: DM

All authors read and approved the final manuscript.

## Pre-publication history

The pre-publication history for this paper can be accessed here:



## Figures and Tables

**Table 1:** Selected avoidable deaths^a^

**Cause of death**	**Intervention**	**Evidence of effectiveness**	**ICD8 code 1969–78**	**ICD9 code 1979–99**	**Age-groups (years)**
***All causes***			000–999	001–999	0–64
Ischaemic heart disease	*Public health * Smoking cessation, lifestyle modification	[21][22][36]	410–413	410–414, 429.2	35–64
	*Medical care * Pharmacotherapy, angioplasty, surgery	[37][38][39][40][41]			
***Deaths amenable to medical care***					
Tuberculosis	Immunization, contact tracing, pharmacotherapy	[42][43]	011–019	010–018, 137	5–64
Malignant neoplasm of the breast [Breast cancer]	Screening, surgery, radiation therapy, chemotherapy	[44][45][46][47]	174	174	25–64
Malignant neoplasm of the cervix uteri [Cervical cancer]	Screening, surgery, radiation therapy, chemotherapy	[48][49]	180	180	15–64
Malignant neoplasm of the uterus (except cervix uteri) [Uterine cancer]	Screening, surgery, radiation therapy, chemotherapy	[50][51]	182	179, 182	15–64
Hodgkin's disease	Chemotherapy, radiation therapy	[52][53][54]	201	201	15–64
Hypertension and cerebrovascular disease	Pharmacotherapy, carotid endarterectomy	[39][55][56][57]	400–404, 430–438	401–405, 430–438	35–64
Asthma	Pharmacotherapy	[58][59]	493	493	5–44
Gastric and duodenal ulcer [Ulcers]	Pharmacotherapy, surgery	[60][61]	531–534	531–534	25–64
Appendicitis	Surgery	[62][63]	540–543	540–543	5–64
Hernia	Surgery	[64]	550–553	550–553	5–64
Cholelithiasis, cholecystitis and cholangitis [Gallbladder diseases]	Pharmacotherapy, surgery	[65]	574–576	575–476	5–64
Complications of pregnancy [Maternal mortality]	Screening, pharmacotherapy, surgery	[66][67]	630–678	630–676	0–64
Perinatal conditions	Screening, pharmacotherapy, surgery	[68]	760–779	760–779	> 28 weeks gestation <1 week and stillbirths
***Deaths amenable to public health***					
Malignant Neoplasm of the Trachea, Bronchus and Lung [Lung cancer]	Smoking cessation, lifestyle modification	[69][70][71]	162	162	35–64
Accidents/Poisonings/Violence [Injuries]	Legislation, safety equipment	[72][73][74][75][76][77][20]	800–999	800–999	0–64

Note – Simplified names for some causes are presented within square brackets.

^a^Adapted from the European Community Working Group on Health Services and "Avoidable Deaths," 1997.[16]

**Table 2:** All-cause mortality under 65 years and 'avoidable' mortality in Canada from 1975 to 1999, males^a, b^

	**1975–1979**		**1980–1984**		**1985–1989**		**1990–1994**		**1995–1999**		
						
**Cause**	**ASMR**	**%**^d^	**ASMR**	**%**	**ASMR**	**%**	**ASMR**	**%**	**ASMR**	**%**	**%change**^e^
All causes	344.5	100.0	292.1	100.0	259.0	100.0	233.4	100.0	203.8	100.0	40.8
											
Injuries	80.1	23.3	67.1	23.0	57.2	22.1	50.9	21.8	46.4	22.8	42.0
Ischaemic heart disease	89.6	26.0	70.7	24.2	54.5	21.0	40.4	17.3	32.2	15.8	64.0
Lung cancer	23.1	6.7	24.9	8.5	24.5	9.5	22.3	9.6	18.1	8.9	21.5
Hypertension and cerebrovascular disease	12.5	3.6	9.3	3.2	7.4	2.9	6.5	2.8	5.4	2.7	56.9
Perinatal conditions	9.2	2.7	6.4	2.2	5.1	2.0	4.2	1.8	3.9	1.9	57.3
Ulcers	1.2	0.3	0.9	0.3	0.7	0.3	0.5	0.2	0.3	0.2	71.7
Hodgkin's disease	0.8	0.2	0.7	0.2	0.6	0.2	0.4	0.2	0.3	0.2	62.3
Asthma	0.2	0.1	0.3	0.1	0.3	0.1	0.2	0.1	0.1	0.1	38.0
Gallbladder diseases	0.4	0.1	0.2	0.1	0.2	0.1	0.1	0.1	0.1	0.1	58.6
Tuberculosis	0.6	0.2	0.4	0.1	0.2	0.1	0.2	0.1	0.1	0.0	84.5
Hernia	0.1	0.0	0.1	0.0	0.1	0.0	0.1	0.0	0.0	0.0	54.7
Appendicitis	0.2	0.0	0.1	0.0	0.1	0.0	0.1	0.0	0.0	0.0	82.1
											
"Avoidable" causes	218.1	63.3	181.1	62.0	150.8	58.2	125.8	53.9	107.2	52.6	50.8
Other causes	126.4	36.7	111.0	38.0	108.1	41.8	107.6	46.1	96.6	47.4	23.6

^a ^The age-groups considered for each cause of death are presented in Table 1.

^b ^Causes are presented from the greatest contribution to all-cause mortality during 1995–1999.

^c ^Age-standardized mortality rate (per 100,000 population).

^d ^Percent of all cause ASMR attributed to each cause.

^e ^Percent change in ASMR from 1975–1979 to 1995–1999.

**Table 3:** All-cause mortality under 65 years and 'avoidable' mortality in Canada from 1975 to 1999, females^a, b^

	**1975–1979**		**1980–1984**		**1985–1989**		**1990–1994**		**1995–1999**		
						
**Cause**	**ASMR**	**%**^d^	**ASMR**	**%**	**ASMR**	**%**	**ASMR**	**%**	**ASMR**	**%**	**%changee**
All causes	175.6	100.0	152.4	100.0	138.5	100.0	126.7	100.0	116.9	100.0	33.4
											
Injuries	27.9	15.9	22.4	14.7	18.9	13.7	16.3	12.9	15.0	12.8	46.3
Breast cancer	16.0	9.1	15.3	10.1	15.7	11.3	14.2	11.2	12.4	10.6	22.5
Lung cancer	6.7	3.8	8.9	5.8	10.9	7.8	11.9	9.4	12.0	10.2	-79.7
Ischaemic heart disease	22.1	12.6	17.9	11.7	14.0	10.1	10.7	8.4	8.7	7.4	60.7
Hypertension and cerebrovascular disease	9.6	5.5	7.4	4.9	5.8	4.2	4.6	3.7	4.2	3.6	56.8
Perinatal mortality	7.1	4.0	4.9	3.2	3.8	2.8	3.4	2.7	3.3	2.8	53.4
Cervical cancer	4.0	2.3	1.1	0.7	2.8	2.0	0.9	0.7	2.4	2.0	41.8
Hodgkin's disease	0.5	0.3	0.4	0.3	0.3	0.2	0.2	0.2	0.2	0.2	61.4
Ulcers	0.4	0.3	0.4	0.3	0.3	0.2	0.2	0.2	0.2	0.1	65.3
Asthma	0.2	0.1	0.3	0.2	0.3	0.2	0.2	0.2	0.1	0.1	37.9
Gallbladder diseases	0.3	0.2	0.2	0.1	0.1	0.1	0.1	0.1	0.1	0.1	57.0
Maternal mortality	0.2	0.1	0.1	0.1	0.1	0.1	0.1	0.1	0.1	0.1	48.2
Tuberculosis	0.3	0.2	0.2	0.1	0.1	0.1	0.1	0.1	0.1	0.1	75.9
Hernia	0.1	0.1	0.1	0.1	0.1	0.0	0.0	0.0	0.1	0.0	54.2
Appendicitis	0.1	0.0	0.0	0.0	0.0	0.0	0.0	0.0	0.0	0.0	57.1
											
"Avoidable" causes	95.5	54.5	79.6	52.2	73.2	52.8	63.1	49.8	58.7	50.2	38.5
Other causes	80.0	45.6	72.8	47.8	65.4	47.2	63.6	50.2	58.2	49.8	27.3

^a^The age-groups considered for each cause of death are presented in Table 1.

^b ^Causes are presented from the greatest contribution to all-cause mortality during 1995–1999.

^c ^Age-standardized mortality rate (per 100,000 population).

^d ^Percent of all cause ASMR attributed to each cause.

^e ^Percent change in ASMR from 1975–1979 to 1995–1999.
